# Cardiac MRI findings to differentiate athlete's heart from hypertrophic (HCM), arrhythmogenic right ventricular (ARVC) and dilated (DCM) cardiomyopathy

**DOI:** 10.1007/s10554-021-02280-6

**Published:** 2021-05-21

**Authors:** J. Kübler, C. Burgstahler, J. M. Brendel, S. Gassenmaier, F. Hagen, K. Klingel, S.-C. Olthof, K. Blume, B. Wolfarth, K. A. L. Mueller, S. Greulich, P. Krumm

**Affiliations:** 1 Department of Radiology, Diagnostic and Interventional Radiology, University of Tübingen, Tübingen, Germany; 2 Department of Internal Medicine V, Sports Medicine, University of Tübingen, Tübingen, Germany; 3 Cardiopathology, Molecular Pathology, University of Tübingen, Tübingen, Germany; 4 Department of Sports Medicine, Humboldt-University/Charité University Medicine, Berlin, Germany; 5 Department of Preventive and Rehabilitative Sports Medicine, Technical University Munich (TUM), Munich, Germany; 6 Department of Internal Medicine III, Cardiology and Cardiovascular Medicine, University of Tübingen, Tübingen, Germany

**Keywords:** DCM, HCM, ARVC, Cardiac MRI, Athlete’s heart

## Abstract

To provide clinically relevant criteria for differentiation between the athlete’s heart and similar appearing hypertrophic (HCM), dilated (DCM), and arrhythmogenic right-ventricular cardiomyopathy (ARVC) in MRI. 40 top-level athletes were prospectively examined with cardiac MR (CMR) in two university centres and compared to retrospectively recruited patients diagnosed with HCM (n = 14), ARVC (n = 18), and DCM (n = 48). Analysed MR imaging parameters in the whole study cohort included morphology, functional parameters and late gadolinium enhancement (LGE). Mean left-ventricular enddiastolic volume index (LVEDVI) was high in athletes (105 ml/m^2^) but significantly lower compared to DCM (132 ml/m^2^; p = 0.001). Mean LV ejection fraction (EF) was 61% in athletes, below normal in 7 (18%) athletes vs. EF 29% in DCM, below normal in 46 (96%) patients (p < 0.0001). Mean RV-EF was 54% in athletes vs. 60% in HCM, 46% in ARVC, and 41% in DCM (p < 0.0001). Mean interventricular myocardial thickness was 10 mm in athletes vs. 12 mm in HCM (p = 0.0005), 9 mm in ARVC, and 9 mm in DCM. LGE was present in 1 (5%) athlete, 8 (57%) HCM, 10 (56%) ARVC, and 21 (44%) DCM patients (p < 0.0001). Healthy athletes’ hearts are characterized by both hypertrophy and dilation, low EF of both ventricles at rest, and increased interventricular septal thickness with a low prevalence of LGE. Differentiation of athlete’s heart from other non-ischemic cardiomyopathies in MRI can be challenging due to a significant overlap of characteristics also seen in HCM, ARVC, and DCM.

**Supplementary Information:**

The online version contains supplementary material available at 10.1007/s10554-021-02280-6.

## Introduction

Highly trained athletes show morphological and functional changes of the cardiovascular system as a response to intensive exercise. The associated process of cardiac remodelling leads to the so called “athlete’s heart” and is considered a physiologic adaptation to repetitively increased volume load and blood pressure [1–3]. However, cardiomyopathies like hypertrophic cardiomyopathy (HCM), arrhythmogenic right ventricular cardiomyopathy (ARVC) or dilated cardiomyopathy (DCM) have sometimes similar alterations. This makes a precise differentiation in individual cases difficult, especially in an early stage of the disease [2, 4, 5]. Since the cardiovascular abnormalities seen in cardiomyopathies are known to be the underlying causes for sudden cardiac death (SCD) it is crucial to differentiate between pathologic findings and physiologic remodelling as in athletes [6–8]. On the one hand, false negative diagnosis and cardiomyopathy patients can lead to progression of disease and delay in therapy. On the other hand, false positive diagnosis in athletes can lead to unnecessary restriction from participation in competitive sports and have significant impact on lifestyle. Cardiac magnetic resonance imaging (CMR) provides an excellent tool to visualize cardiac pathologies and assess morphological and functional parameters [9].

The aim of this multicentre study was to provide clinically relevant criteria to help differentiate between the athlete’s heart and cardiomyopathies.

## Material and methods

### Athletes

We included 40 German Caucasian top-level athletes participating in top national and international competitions, mainly endurance athletes, who were prospectively examined for this multicentre trial. In 19 athletes CMR was performed at the University Hospital Tübingen (Tübingen Germany), and in 21 CMR was performed at the University Hospital of the Technical University Munich (Munich, Germany).

The study protocol was designed according to ethical standards in Sport and Exercise Science Research.

### Patients

Patients who underwent a CMR between 2008 and 2019 and were diagnosed with HCM (n = 14), ARVC (n = 18) or DCM (n = 48) were included retrospectively. Diagnosis of DCM was proven by endomyocardial biopsy in all patients, the diagnosis of ARVC in nine patients respectively. Endomyocardial biopsies (EMB) and histopathologic workup was performed as previously described [10].

HCM patients were clinically diagnosed with HCM in the Sports Medicine Clinic in Tübingen. Patients with additional alternative cardiac diagnosis in CMR or EMB were excluded.

### Ethics approval

The study protocol was approved by the Institutional Review Board (IRB) of the University of Tübingen (reference centre; 315/2011BO2).

### Image acquisition

CMR was performed using a 1.5-T system (Magnetom Aera or Avanto, Siemens Healthcare, Erlangen, Germany). Sequences were ECG-triggered and performed in breath hold technique using a body array coil as previously described [11, 12].

Myocardial function was assessed with cine steady state free precession (SSFP) loops that were acquired in four chamber view (4CV), two chamber view (2CV) in both ventricles, three chamber view (3CV), and a stack of short axis (SA) slices covering both ventricles from base to apex.

Late Gadolinium Enhancement (LGE) imaging was performed with 2D inversion recovery gradient echo sequences acquired in 4CV, 2CV and a stack of SA views 10 min after intravenous administration of contrast agent Gadobutrol (Gadovist, Bayer Healthcare, Leverkusen, Germany) at a dosage of 0.15 mmol/kg body weight. To suppress signal from healthy myocardium, an inversion time localizer was used, to determine the optimal inversion time (TI scout). The inversion time was adjusted individually to 260–340 ms, to minimize signal from normal myocardium.

### Image analysis

Analysis of CMR images was performed by two readers in consensus at an offline workstation using cmr42 (Circle Cardiovascular Imaging, Calgary AB, Canada).

End-diastolic LV myocardial thickness was measured using a mid-ventricular short-axis slice at the interventricular septum (IVS) and infero-lateral wall (ILW). LV end-diastolic diameter (LVEDD), RV end-diastolic diameter (RVEDD), and RV myocardial thickness were measured at the inferior wall in the same image. Left and right atrial sizes were quantified using planimetry in 4CV as previously described [13].

For functional analysis of left and right ventricle, endocardial (both ventricles) and epicardial (only LV) contours were semi-automatically drawn and carefully adjusted manually: Left and right ventricular end-diastolic volume (LVEDV, RVEDV), end-systolic volume (LVESV, RVESV), stroke volume (LVSV, RVSV), ejection fraction (LVEF, RVEF), and myocardial mass (LVMM). Left ventricular global function index (LVGFI) in % was calculated according to the equation introduced by Mewton et al., and myocardial density is defined as 1.05 g/ml [14]:

$$LVGFI = \frac{LVSV}{{\frac{{\left( {LVEDV + LVESV} \right)}}{2} + \frac{LVMM}{{Myocardial\_density}}}}*100$$

LV-remodelling index was calculated as the ratio between indexed LV myocardial mass and indexed LV end-diastolic volume [15].

Body surface normalization was applied to determine index values and calculated on the basis of height and weight by using the Mosteller method. Assessed morphological and functional parameters were compared to normal reference values as published by Kawel-Boehm et al. and Hergan et al. [16, 17].

LGE imaging was evaluated visually according to the recommendations of the Society for Cardiovascular Magnetic Resonance task force [18]. Image contrast and brightness was modified to minimize background signal.

### Statistical analysis

Statistical analysis was performed using JMP (Version 14.2.0, SAS Institute Inc., Cary NC, USA) and SPSS 22.0 (IBM Corp., Armonk, NY, USA). Continuous variables are expressed as mean value ± standard deviation (SD). Range is given in square brackets. Normal distribution of functional parameters was assessed visually in curves using Saphiro–Wilk test [19]. Two-sided t-tests on paired differences were applied for normally distributed variables, for non-normally distributed variables a Wilcoxon rank sum test was used. αglob = 0.05 was chosen as the global level of significance (αglob). 66 tests were performed (k = 66). Local level of significance (αloc) for each test on dependent variables was corrected according to the Bonferroni equation αloc = αglob/k = 0.00075. Statistical tests between groups were performed with regard to athletes since the aim of this study is to provide criteria for differentiation between athlete’s hearts and cardiomyopathies.

## Results

### Athletes’ characteristics and CMR

**Fig. 1:**
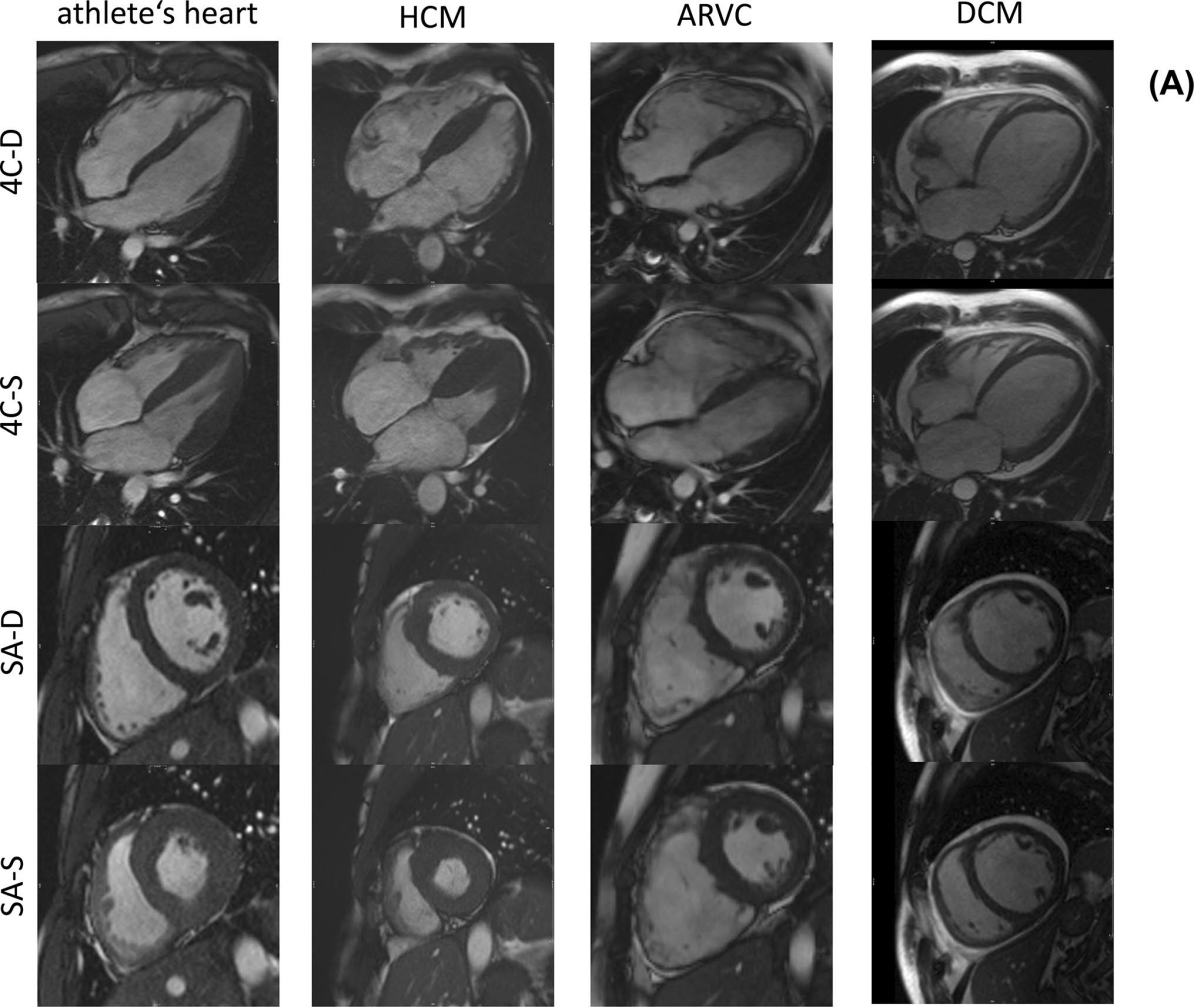

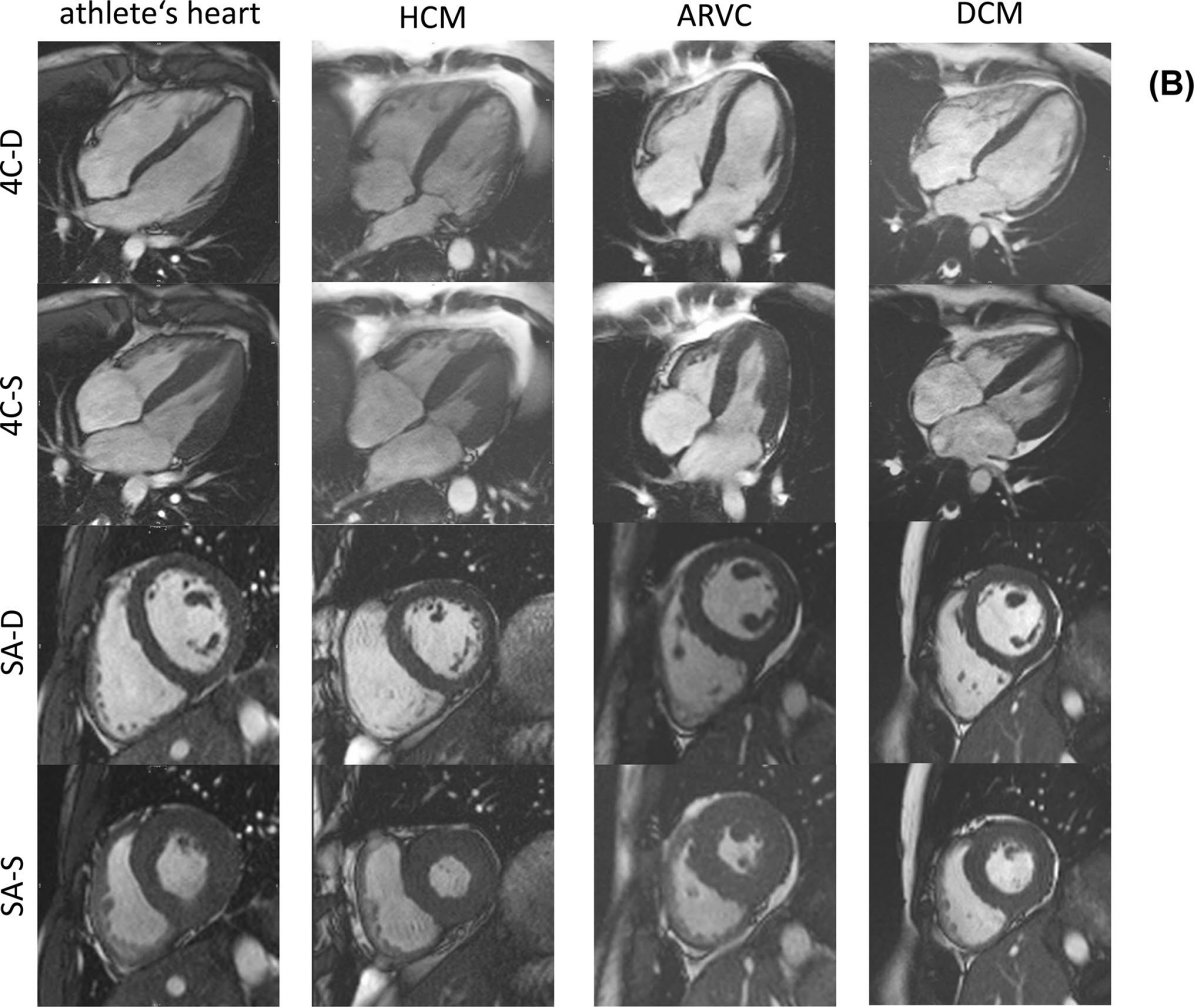
**a** Characteristic CMR examples of an athlete’s heart and cardiomyopathies. Typical elliptic LV shape of an athlete’s heart in SA. **b** CMR examples of athlete’s heart and cardiomyopathies with less distinctive imaging features. *HCM* hypertrophic cardiomyopathy; *ARVC* arrhythmogenic right ventricular cardiomyopathy; *DCM* dilated cardiomyopathy; *4C-D* enddiastolic four chamber view; *4C-S* endsystolic four chamber view; *SA-D* enddiastolic short axis view; *SA-S* endsystolic short axis view

Athlete’s characteristics are summarized in Tables 1 and 2, morphological and functional parameters of athletes and patients in Table 3. ECG abnormalities were analysed according to Seattle Criteria [20]. Athletes were young (age 24 ± 4 years) and predominantly male (68%). Most athletes were endurance athletes performing triathlon (22.5%), athletics (22.5%), and cycling (15%). Mean BMI was 21.9 ± 1.2 kg/m^2^. Most athletes (82%) had normal LVEF (61% ± 5). LVSVI was within reference range in all subjects (63 ± 9 ml/m^2^). LVEDVI and RVEDVI were elevated in 58% and 50% of athletes (105 ± 17 ml/m^2^ and 118 ± 21 ml/m^2^, respectively). RVEF was reduced in 40% of athletes (54 ± 5 ml). The majority of athletes (68%) showed increased RVEDD (49 ± 8 mm). Indexed LV myocardial mass was elevated in 40% of athletes (84 ± 22 g/m^2^). Interventricular myocardial thickness was elevated beyond normal range in one athlete (10 ± 2 mm) and inferolateral wall thickness was elevated in 2 athletes (8 ± 2 mm). Cine-sequences demonstrated no kinetic disorders. Septal linear mid-myocardial LGE was present in one athlete. A representative example of an athlete’s heart is demonstrated in Fig. 1.

**Table 1:** Characteristics of athletes and patients

	Athletes	DCM	ARVC	HCM
	n = 40	n = 48	n = 18	n = 14
Age—years	24 ± 4	56 ± 12	37 ± 15	44 ± 17
Sex male—n (%)	27 (68%)	39 (81%)	11 (61%)	14 (100%)
Weight—kg	71 ± 11	87 ± 19	79 ± 19	80 ± 8
Heigth—cm	180 ± 10	176 ± 7	175 ± 13	178 ± 6
BSA—m^2^	1.88 ± 0.19	2.04 ± 0.24	1.95 ± 0.29	1.99 ± 0.13
BMI—kg/m^2^	21.9 ± 1.2	28.0 ± 6.0	25.3 ± 3.8	25.2 ± 1.8
ECG abnormalities—n (%)	9 (23%)	16 (33%)	9 (50%)	10 (71%)
NYHA classification				
NYHA 1	–	7 (15%)	14 (78%)	11 (79%)
NYHA 2	–	16 (33%)	4 (22%)	3 (21%)
NYHA 3	–	23 (48%)	0	0
NYHA 4	–	2 (4%)	0	0
Sports—n (%)		0	0	0
Triathlon	9 (22.5%)			
Athletics	9 (22.5%)			
Cycling	6 (15%)			
Biathlon	4 (10%)			
Volleyball	4 (10%)			
Skiing	2 (5%)			
Other	6 (15%)			

Values are presented as mean ± SD

*BSA* body surface area; *BMI* body mass index; *ECG* electrocardiogram; *n/a* data not available; *NYHA* New York Heart Association classification of heart failure

**Table 2:** Individual morphological and functional characteristics in athletes

Athlete no.	Age	Sex	LV enlargement			LV-EF reduced	RV enlargement			RV-EF reduced	Spetal myocardial thickness elevated	kinetic disorder	LGE presence	Intraindividual sum of criterions
			EDV	ESV	EDD		EDV	ESV	EDD					
1	30	M	**Yes**	**Yes**	No	No	**Yes**	**Yes**	No	**Yes**	**Yes**	No	No	6
2	29	F	No	No	No	No	**Yes**	**Yes**	**Yes**	**Yes**	No	No	No	4
3	30	M	No	**Yes**	No	No	No	**Yes**	No	No	**Yes**	No	No	3
4	32	M	**Yes**	**Yes**	No	No	**Yes**	**Yes**	No	No	**Yes**	No	No	5
5	24	F	**Yes**	**Yes**	No	No	**Yes**	**Yes**	**Yes**	No	No	No	No	5
6	22	M	**Yes**	**Yes**	No	No	**Yes**	**Yes**	**Yes**	No	No	No	No	5
7	29	F	No	No	No	No	**Yes**	**Yes**	**Yes**	No	No	No	No	3
8	27	F	**Yes**	**Yes**	**Yes**	No	**Yes**	**Yes**	**Yes**	No	No	No	No	6
9	23	F	**Yes**	**Yes**	**Yes**	No	**Yes**	**Yes**	**Yes**	No	No	No	No	6
10	18	M	No	No	No	No	**Yes**	**Yes**	**Yes**	No	No	No	No	3
11	19	M	**Yes**	**Yes**	No	No	**Yes**	**Yes**	**Yes**	**Yes**	No	No	No	6
12	27	M	**Yes**	**Yes**	No	No	**Yes**	**Yes**	**Yes**	No	No	No	No	5
13	26	M	**Yes**	**Yes**	**Yes**	No	**Yes**	**Yes**	**Yes**	**Yes**	No	No	No	7
14	23	M	**Yes**	No	No	No	No	No	No	No	No	No	No	1
15	21	M	No	**Yes**	No	No	**Yes**	**Yes**	**Yes**	No	No	No	No	4
16	21	M	**Yes**	No	No	No	No	No	**Yes**	No	No	No	No	2
17	18	F	No	No	No	No	**Yes**	**Yes**	**Yes**	No	No	No	**Yes**	4
18	19	M	No	No	No	No	No	No	No	No	No	No	No	0
19	21	M	**Yes**	**Yes**	No	**Yes**	**Yes**	No	No	No	No	No	No	4
20	24	M	**Yes**	**Yes**	No	No	**Yes**	**Yes**	**Yes**	No	No	No	No	5
21	20	F	No	No	No	No	No	**Yes**	No	No	No	No	No	1
22	23	M	**Yes**	**Yes**	No	**Yes**	**Yes**	**Yes**	**Yes**	**Yes**	No	No	No	7
23	25	M	**Yes**	**Yes**	No	**Yes**	**Yes**	**Yes**	No	No	No	No	No	5
24	20	F	No	No	No	No	No	No	**Yes**	No	**Yes**	No	No	2
25	22	M	**Yes**	**Yes**	No	**Yes**	**Yes**	**Yes**	**Yes**	**Yes**	No	No	No	7
26	20	F	No	No	No	No	No	**Yes**	**Yes**	**Yes**	No	No	No	3
27	20	M	**Yes**	**Yes**	No	No	No	**Yes**	No	No	**Yes**	No	No	4
28	26	M	**Yes**	No	No	No	**Yes**	**Yes**	**Yes**	No	**Yes**	No	No	5
29	29	M	**Yes**	**Yes**	No	No	**Yes**	**Yes**	**Yes**	No	**Yes**	No	No	6
30	33	M	**Yes**	No	No	No	**Yes**	**Yes**	**Yes**	No	**Yes**	No	No	5
31	29	M	**Yes**	**Yes**	No	**Yes**	**Yes**	**Yes**	**Yes**	**Yes**	**Yes**	No	No	8
32	26	M	**Yes**	**Yes**	No	**Yes**	**Yes**	**Yes**	**Yes**	No	**Yes**	No	No	7
33	31	M	**Yes**	**Yes**	No	**Yes**	**Yes**	**Yes**	**Yes**	**Yes**	**Yes**	No	No	8
34	31	M	**Yes**	**Yes**	No	No	**Yes**	**Yes**	No	No	**Yes**	No	No	5
35	22	M	No	No	No	No	No	No	No	No	No	No	No	0
36	19	F	No	No	No	No	**Yes**	No	**Yes**	No	No	No	No	2
37	26	M	**Yes**	**Yes**	No	No	**Yes**	**Yes**	**Yes**	No	No	No	No	5
38	30	F	No	**Yes**	No	No	No	No	**Yes**	No	No	No	No	2
39	23	F	No	No	No	No	No	No	No	No	No	No	No	0
40	17	F	**Yes**	**Yes**	No	No	**Yes**	**Yes**	No	No	**Yes**	No	No	5
Sum criterion over all athletes—n (%)			26 (65%)	25 (63%)	3 (8%)	7 (18%)	29 (73%)	31 (78%)	27 (68%)	9 (23%)	13 (33%)	0	1 (3%)	

Parameters were evaluated according to age and gender adjusted reference values [16]

*EDV* end-diastolic volume, *ESV* end-systolic volume, *EF* ejection fraction, *EDD* end-diastolic diameter, *LV* left ventricle, *RV* right ventricle, *LGE* late gadolinium enhancement

The abnormal findings ("yes") are marked in bold

**Table 3:** Morphological and functional parameters of athletes and patients

	Athletes n = 40	HCM n = 14	ARVC n = 18	DCM n = 48	p-value athletes vs. HCM	p-value athletes vs. ARVC	p-value athletes vs. DCM
Age—years	**24 ± 4**	**44 ± 17**	**37 ± 15**	**55 ± 12**	**0.0001**	**0.0009**	** < 0.0001**
Sex male—n (%)	**27 (68%)**	**14 (100%)**	**11 (61%)**	**39 (81%)**			
LV-parameters							
LVEDVI [ml/m^2^]	**105 ± 17**	**94 ± 13**	**89 ± 22**	**132 ± 41**	**0.024**	**0.009**	**0.001**
Elevated—n (%)	23 (58%)	4 (29%)	5 (28%)	34 (71%)			
LVESVI [ml/m^2^]	**41 ± 9**	**39 ± 9**	**42 ± 17**	**96 ± 40**	0.37	0.6	** < 0.0001**
Elevated—n (%)	29 (73%)	7 (50%)	11 (61%)	46 (96%)			
LVSVI [ml/m^2^]	**63 ± 9**	**55 ± 11**	**47 ± 13**	**36 ± 13**	**0.017**	** < 0.0001**	** < 0.0001**
Reduced—n (%)	0	2 (14%)	7 (39%)	33 (69%)			
LVEF [%]	**61 ± 5**	**59 ± 9**	**54 ± 11**	**29 ± 13**	0.61	**0.001**	** < 0.0001**
Reduced—n (%)	7 (18%)	5 (36%)	11 (61%)	46 (96%)			
LVMMI [g/m^2^]	**84 ± 22**	**77 ± 12**	**57 ± 14**	**70 ± 21**	**0.47**	** < 0.0001**	**0.005**
Elevated—n (%)	16 (40%)	3 (21%)	0	9 (19%)			
LVEDD [mm]	**53 ± 5**	**54 ± 5**	**52 ± 8**	**67 ± 8**	0.55	0.53	** < 0.0001**
Elevated—n (%)	6 (15%)	1 (7%)	2 (11%)	38 (79%)			
LVGFI	**42 ± 7**	**40 ± 9**	**41 ± 12**	**22 ± 9**	0.57	0.41	** < 0.0001**
LV-Remodelling Index	**0.8 ± 0.16**	**0.83 ± 0.14**	**0.66 ± 0.17**	**0.55 ± 0.14**	0.73	**0.002**	** < 0.0001**
RV-parameters							
RVEDVI [ml/m^2^]	**118 ± 21**	**98 ± 16**	**103 ± 26**	**90 ± 25**	**0.0014**	**0.036**	** < 0.0001**
Elevated—n (%)	20 (50%)	1 (7%)	6 (33%)	6 (13%)			
RVESVI [ml/m^2^]	**55 ± 15**	**39 ± 11**	**56 ± 23**	**54 ± 24**	**0.0007**	0.71	0.35
Elevated—n (%)	18 (45%)	1 (7%)	8 (44%)	17 (35%)			
RVSVI[ml/m^2^]	**63 ± 9**	**58 ± 8**	**47 ± 13**	**36 ± 13**	0.08	** < 0.0001**	** < 0.0001**
Reduced—n (%)	0	1 (7%)	6 (33%)	33 (69%)			
RVEF [%]	**54 ± 5**	**60 ± 6**	**46 ± 10**	**41 ± 14**	**0.0013**	**0.0015**	** < 0.0001**
Reduced—n (%)	16 (40%)	2 (14%)	13 (72%)	36 (75%)			
RVEDD [mm]	**49 ± 8**	**51 ± 6**	**48 ± 15**	**46 ± 8**	0.32	0.75	0.08
Elevated—n (%)	27 (68%)	10 (71%)	13 (72%)	21 (44%)			
LVEDV/RVEDV-ratio	**0.89 ± 0.08**	**0.99 ± 0.18**	**0.89 ± 0.22**	**1.5 ± 0.42**	**0.03**	0.27	** < 0.0001**
Myocardial thickness IVS (mm)	**9.7 ± 1.7**	**12.4 ± 2.4**	**9.2 ± 1.8**	**8.8 ± 1.8**	**0.0005**	0.35	**0.044**
Elevated—n (%)	1 (4%)	6 (43%)	0	0			
Myocardial thickness ILW (mm)	**8.4 ± 1.6**	**8.4 ± 1.4**	**7.2 ± 2.6**	**6.5 ± 1.6**	0.9	**0.037**	** < 0.0001**
Elevated—n (%)	2 (5%)	0	1 (6%)	1 (2%)			
Myocardial thickness RV (mm)	**3.7 ± 0.6**	**2.4 ± 0.8**	**2.0 ± 0.8**	**2.0 ± 0.6**	** < 0.0001**	** < 0.0001**	** < 0.0001**
Elevated—n (%)	0	0	0	0			
LAI (cm^2^)	**13 ± 2**	**12 ± 2**	**9 ± 2**	**13 ± 3**	0.53	**0.0002**	0.38
Elevated—n (%)	7 (18%)	0	0	11 (23%)			
RAI (cm^2^)	**14 ± 4**	**13 ± 2**	**11 ± 3**	**12 ± 3**	0.8	**0.021**	**0.036**
Elevated—n (%)	6 (15%)	2 (14%)	1 (6%)	7 (15%)			
LV kinetic disorder—n (%)	**0**	**5 (36%)**	**7 (39%)**	**36 (75%)**			
LV dyssynchrony	0	1 (7%)	1 (6%)	7 (14%)			
LV hypokinesia	0	5 (36%)	7 (39%)	33 (69%)			
LV akinesia	0	1 (7%)	0	12 (25%)			
RV kinetic disorder—n (%)	**0**	**0**	**11 (61%)**	**3 (6%)**			
RV dyssynchrony	0	0	3 (17%)	1 (2%)			
RV hypokinesia	0	0	11 (61%)	4 (4%)			
RV akinesia	0	0	3 (17%)	0			
LGE presence—n (%)	**1 (5%)**	**8 (57%)**	**10 (56%)**	**21 (44%)**			
LGE location—n (%)							
Subepicardial linear/patchy	1 (5%)	4 (29%)	2 (11%)	20 (42%)			
Midwall		4 (29%)	1 (6%)	3 (6%)			
Subendocardial	0	0	0	0			
RV insertion	0	0	0	0			
RV involvement	0	0	7 (39%)	0			

Defined significance level are marked in bold

Mean values ± standard deviations are tabulated

*ARVC* arrhythmogenic right ventricular cardiomyopathy, *HCM* hypertrophic cardiomyopathy, *DCM* dilated cardiomyopathy, *LVEDV* left ventricular end-diastolic volume, *LVESV* left ventricular end-systolic volume, *LVSV* left ventricular stroke volume, *LVEF* left ventricular ejection fraction, *LVMM* left ventricular myocardial mass, *LVEDVI* left ventricular end-diastolic volume index, *LVMMI* left ventricular myocardial mass index, *LVEDD* left ventricular end-diastolic diameter, *IVS* interventricular septum, *ILW* infero-lateral wall, *LVGFI* left ventricular global function index, *WMA* wall motion abnormalities, *RVEDV* right ventricular end-diastolic volume, *RVESV* right ventricular end-systolic volume, *RVSV* right ventricular stroke volume, *RVEF* right ventricular ejection fraction, *RVEDVI* right ventricular end-diastolic volume index, *LA* left atrium, *LAI* left atrium index, *RA* right atrium, *RAI* right atrium index, *LGE* late gadolinium enhancement, *CI* confidence interval

### HCM

**Fig. 2:**
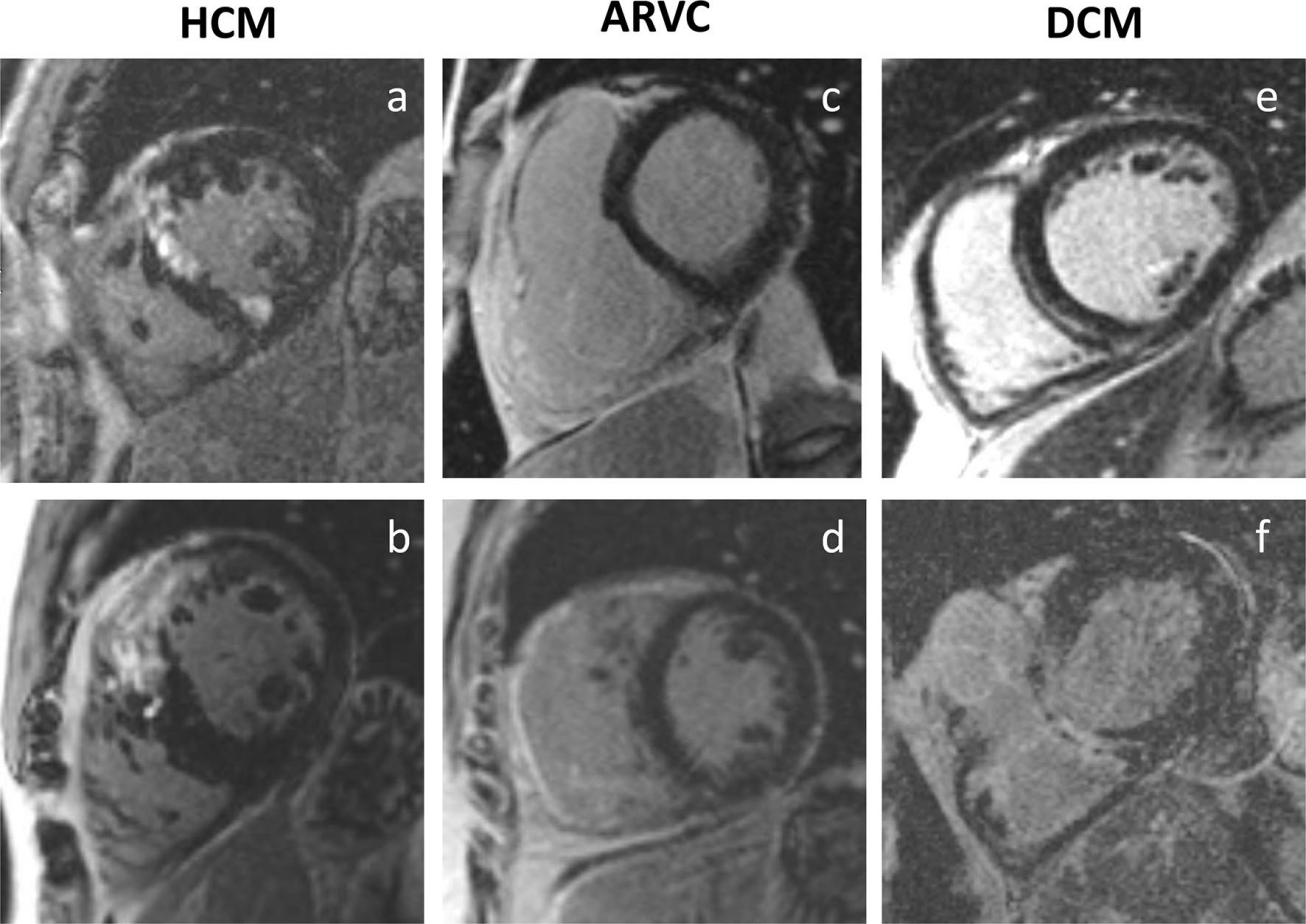
Examples of late gadolinium enhancement (LGE) in six representative patients. Examples of late gadolinium enhancement (LGE) in six representative patients. Hypertrophic cardiomyopathy (HCM, **a**, **b**) shows intensive LGE of the interventricular septum. In arrhythmogenic right ventricular cardiomyopathy (ARVC, **c**, **d**) extensive LGE of the entire right ventricle may be found (**d**) but is no necessary feature for ARVC diagnosis. Intramural septal LGE of dilated left ventricles in patients with dilated cardiomyopathy (DCM, **e**, **f**)

Athletic HCM patients were 44 ± 17 years, all male. Reduced left ventricular function was observed in 36% of HCM patients, compared to healthy athletes there was no statistically significant difference (59 ± 9% vs. 61 ± 5%, p = 0.6). LVMMI was similar (77 ± 12 vs. 84 ± 22 g/m^2^, p = 0.47) but thickness of the interventricular septum was significantly higher in HCM patients compared to athletes (12 ± 2 mm vs. 10 ± 2 mm, p = 0.0005; representative example in Fig. 1) whereas the thickness of the right ventricular myocardium was significantly higher in athletes (4 ± 0.6 vs. 2 ± 0.8 mm, p < 0.0001). LV-remodelling index was higher than in all other groups, but not significantly different from athletes (0.83 ± 0.14 vs. 0.8 ± 0.16, p = 0.73). Wall motion abnormalities could be observed in 36% of HCM patients, predominantly showing hypokinesia. LGE was present in 57% of HCM patients, mainly subepicardial (29%) and mid-myocardial (29%, Fig. 2).

### Characteristics in ARVC

Of the 18 ARVC patients, mean aged 37 ± 15 years, 61% were male. LVEF was reduced in 11 patients of this group (61%) and significantly lower than in athletes (54 ± 11% vs. 61 ± 5%, p = 0.001), LVSVI was reduced in 39% (47 ± 13 ml/m^2^, p < 0.0001). RVEF was reduced in 72% of ARVC patients and significantly lower than in athletes (46 ± 10% vs. 54 ± 5%, p = 0.0015). RVEDVI was elevated beyond reference range in 33%, but lower in ARVC patients than in athletes (103 ± 26 vs. 118 ± 21 ml/m^2^, p = 0.036). LVEDV/RVEDV ratio was very similar to athletes (0.89 ± 0.22 vs. 0.89 ± 0.08). Kinetic disorders, especially hypokinesia, were common in ARVC patients affecting both the right and the left ventricle (61% and 39%, respectively). The majority of ARVC patients (56%) showed LGE, mostly involving the right ventricle (39%, Fig. 2).

### Characteristics in DCM

Patients with biopsy proven DCM (mean age 55 ± 12 years, 81% male) were almost all (96%) characterized by reduced LVEF (29 ± 13%). 71% showed increased LVEDVI (132 ± 41 ml/m^2^, p = 0.001) and 96% increased LVESVI (96 ± 40 ml/m^2^), both significantly higher compared to athletes (p = 0.001 and p < 0.0001, respectively). LVEDD was elevated in 79% and significantly larger than in athletes (LVEDD: 67 ± 8 vs. 53 ± 5 mm, p < 0.0001). RVEDVI was normal in most DCM patients (90 ± 25 ml/m^2^), but RVEF was reduced in 75% (41 ± 14%). The ratio of LVEDV/RVEDV was significantly higher compared to athletes (1.5 ± 0.42 vs. 0.89 ± 0.08, p < 0.0001). LVGFI and LV-remodelling index were both lowest among all groups and significantly different from athletes (LVGFI: 22 ± 9 vs. 42 ± 7, p < 0.0001; LV-remodelling index: 0.55 ± 0.14 vs. 0.8 ± 0.16, p < 0.0001). 75% showed kinetic disorders of the left ventricle and some patients (6%) also dyskinesia of the right ventricle. LGE was present in almost half of patients and predominantly subepicardial in a linear or patchy pattern (42%, Fig. 2).

**Fig. 3:**
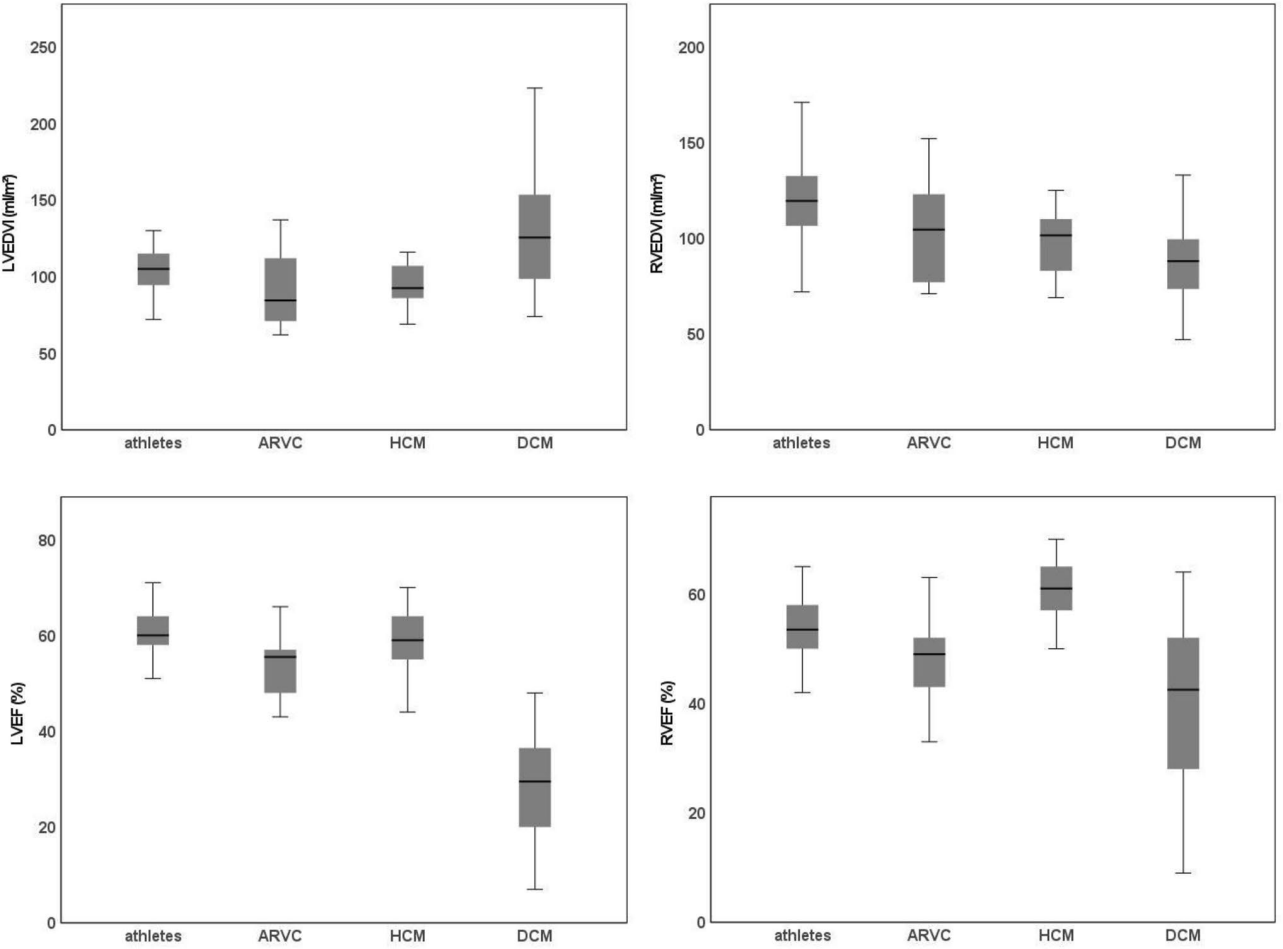
Functional LV and RV parameters of all groups. *HCM* hypertrophic cardiomyopathy; *ARVC* arrhythmogenic right ventricular cardiomyopathy; *DCM* dilated cardiomyopathy; *LVEDVI* left ventricular end-diastolic volume index; *RVEDVI* right ventricular end-diastolic volume index; *RVEF* right ventricular ejection fraction; *LVE*F left ventricular ejection fraction

**Fig. 4:**
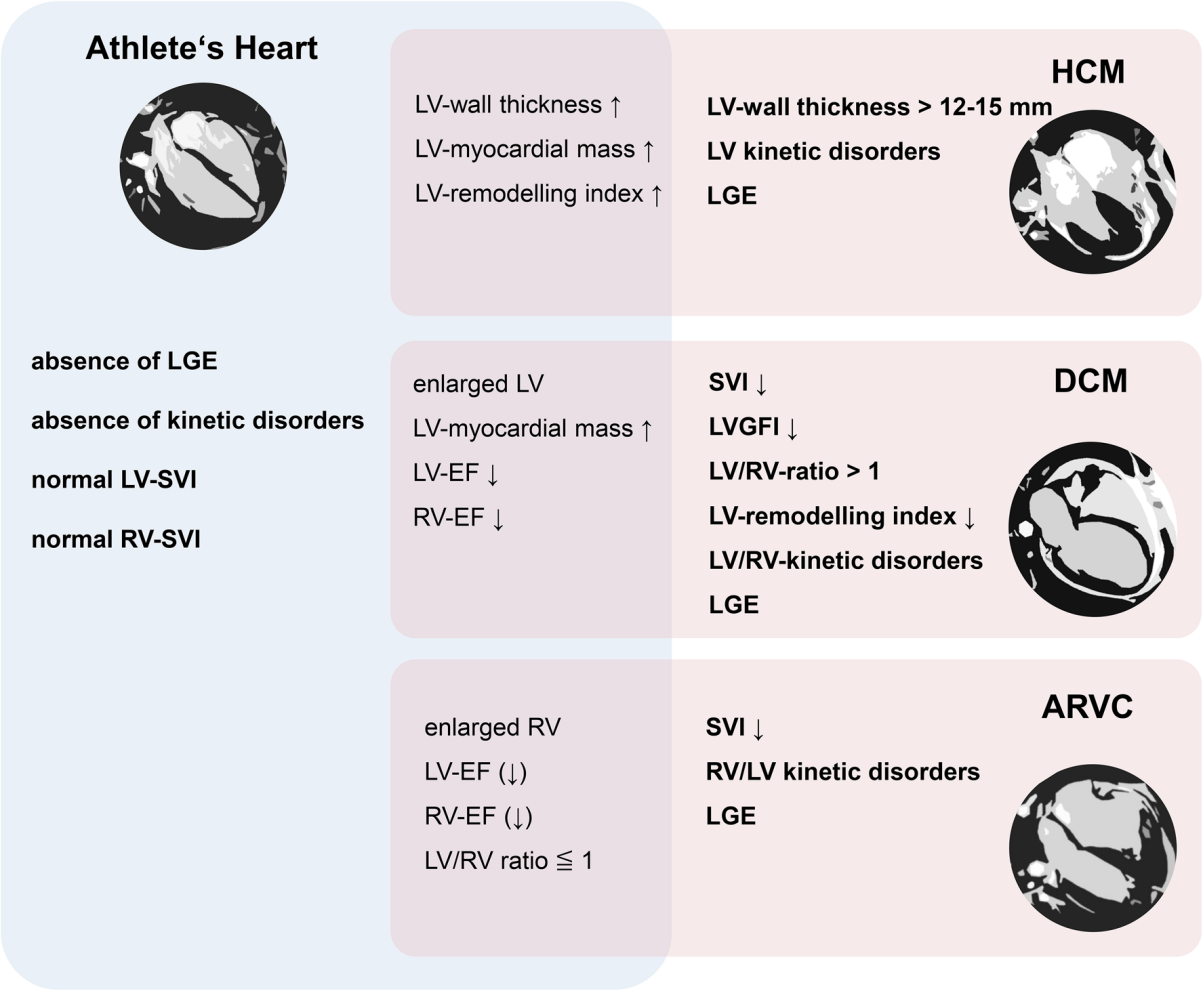
Parameters in favour of athlete’s heart, cardiomyopathies or both. *LV* left ventricle; *RV* right ventricle; *SVI* indexed stroke volume; *EF* ejection fraction; *LGE* late gadolinium enhancement; *LVGFI* left ventricular global functional index; *ARVC* arrhythmogenic right ventricular cardiomyopathy; *HCM* hypertrophic cardiomyopathy; *DCM* dilated cardiomyopathy

Functional LV and RV parameters of all groups are plotted for comparison in Fig. 3. Characteristics and overlaps of groups are visualized in Fig. 4. Exemplary histopathological images of endomyocardial biopsies in ARVC and DCM are presented in Fig. 5 (Supplemental).

## Discussion

In this multicentre study, we compared morphologic and functional CMR parameters of national top-level athletes and patients with HCM, ARVC, and DCM to establish relevant diagnostic imaging criteria for disease differentiation.

While recognition of pathologic findings can be obvious in untrained individuals, differentiation of cardiomyopathies from trained athletes’ hearts may be much more challenging due to considerable overlap between physiologic and pathologic remodelling. Notwithstanding, classifying the athlete’s heart as a “healthy” condition is still at dispute and the positive effects of moderate exercise on cardiovascular risk in general community cannot be transferred to top-level athletes [21].

### Functional parameters

The process of remodelling in athletes is caused by repetitive increased volume and pressure load which eventually leads to hypertrophy and enlargement of all cavities [12]. This circumstance limits the use of mere dimension based parameters for differentiation between the other cardiac impairments [22].

In our study, both indexed EDV of the left and right ventricle were increased in more than half of athletes. Enlarged EDVI could also be observed in DCM patients but was more distinctive and in the majority of patients (78%) linked with significantly increased LVEDD above average.

Enlargement of the right ventricle was common in ARVC patients. RVEDVI > 110 ml/m^2^ (male) or > 100 ml/m^2^ (female) in CMR are major ARVC task force criteria when combined with wall motion abnormalities [23]. However, most athletes showed increase in RVEDVI with values even larger than in ARVC. The dilatation of the right ventricle is also represented by increased RVEDD similarly in athletes and ARVC patients. Yet, in contrast to athletes, enlargement of ventricles was mostly asymmetric in cardiomyopathies with predominantly large LV-volumes in DCM patients and large RV-volumes in ARVC patients. In DCM patients, this fact was respectively mirrored by increased LVEDV/RVEDV-ratio greater than one and significantly higher than in athletes as a helpful parameter for DCM diagnosis. Likewise, LVGFI was relevantly reduced in DCM without overlaps to the other groups, to further flag DCM.

In contrast LVGFI and LV-remodelling index were not helpful to differentiate athlete’s hearts from HCM or ARVC. LVGFI had previously failed in differentiation of cardiac function in different groups of chronic coronary syndrome with and without myocardial infarction but seems to be most sensitive to detect DCM in our study [24]. The inclusion of myocardial mass in LVGFI seems to intermingle volumetric results with morphology in a scale with only small differences between physiologic and pathologic. Thus, clinical use of LVGFI is rather unusual.

Balanced enlargement of the left and right ventricle, which can be observed in most athletes, had already been described previously as a physiologic adaptation attributed to symmetric volume load in endurance sports [1, 25]. Ejection fraction of the left ventricle was low-normal in most athletes but some showed mildly reduced RVEF. Both slight reduction in left and right ventricular function are not uncommon among highly trained endurance athletes [26, 27].

A significantly reduced LV and RV function were observed both in ARVC and DCM. Reduction of RVEF below 45 or 40% is an alternative ARVC task force criterion to RV dilatation in CMR when combined with wall motion abnormalities [23]. As expected, in HCM, we did not observe a significant reduction of LV or RV function. [28]. Even in athletes with reduced EF, indexed stroke volume was within reference range. Especially in subjects with large EDV and borderline EF, normal SV might help unmask physiologic remodelling.

Cine sequences in CMR allow sensitive detection and precise localization of kinetic disorders. Wall motion abnormalities, mainly hypokinesia and akinesia, were common in all cardiomyopathies, but not present in any of the athletes.

### Morphology and viability

An increase in LV myocardial mass was observed in athletes, HCM, and DCM patients.

Athletes show LV hypertrophy as a physiologic response to training [1]. HCM patients may mimic this feature, demonstrating increased LV wall thickness with preponderance of the insertion points. Yet, only one athlete showed hypertrophy of the interventricular septum above 12 mm. Thickness of the septal wall up to 15 mm may be present in up to 2% of highly trained athletes [25, 29–32]. Increase of LV myocardial mass in DCM seems contradictory initially, but ventricular dilatation results in cardiomegaly and increased LV myocardial mass.

LGE is a valuable but non-specific imaging technique for identification of myocardial damage [33] which was present in the majority of our patients. DCM and HCM patients predominantly showed subepicardial or mid-myocardial LGE pattern, whereas ARVC patients were characterized by involvement of the RV. Allocation was therefore dependent on the character of cardiomyopathy exposing fibrotic tissue caused by pathologic remodelling. One athlete showed septal mid-myocardial LGE of the septum, rather suggesting DCM initially. However, CMR follow-up after two years demonstrated stable intramural LGE, without progressive enlargement of ventricles. Therefore, the diagnosis of DCM was rejected and the LGE was attributed to myocarditis. The overall prevalence of focal LGE confined to the hinge points in highly trained endurance athletes has recently been reported relevantly higher compared to control subjects due to focal fibrosis [34]. In our athletes’ cohort, we could not find any LGE at the RV insertions. Generally, LGE is considered as a pathologic finding both in athletes and patients with cardiomyopathies.

The detection of LGE in cardiomyopathies is known to be associated with increased risk for sudden cardiac death [35]. Therefore, a lack of LGE and normal wall motion favours athlete’s heart, whereas presence of LGE and wall motion abnormalities suggests an underlying pathology.

Our study has several limitations. Only Caucasian national top-level athletes were examined. Whereas athletes were investigated prospectively, cardiomyopathy patients were retrospectively enrolled. There is a significant difference in age between athletes and cardiomyopathy patients, with most patients being older than athletes. We addressed this issue in applying age-adjusted reference values for comparison between groups. Furthermore, this study investigates only one time point in athletes and dynamic changes could not be included as parameters for differentiation. Clinically, in indistinct cases repetition of a scan after a period of time may provide further information. Whereas cardiomyopathies show deterioration without treatment, detraining effects can be a characteristic feature in athletes [36]. In addition, no clinical data or ECG findings or family history would be included in the evaluation, which is standard in clinical routine. However, this information is often not available to the physician who performed the CMR examination, so that the present work is primarily to be understood as guidance for the evaluation of the pure CMR data.

## Conclusion

Healthy highly-trained athlete hearts are characterized by: (1) a balanced hypertrophy and dilation, and (2) low EF of both ventricles, (3) (slightly) increased interventricular septal thickness, and (4) increased LV-remodelling index. Differentiation of athlete’s heart from other cardiomyopathies can be challenging due to significant overlap in features of HCM, ARVC, and DCM.

However, both absences of kinetic disorders or LGE as well as normal indexed SV are representative for athlete hearts.

## Supplementary Information

Below is the link to the electronic supplementary material.

Supplementary file1 (PPTX 442 kb)
Figure 5 (Supplemental): Visualization of myocardial damage in endomyocardial biopsies by Masson Trichrome staining. (A) Arrhythmogenic right ventricular cardiomyopathy, × 100, (B) Dilated cardiomyopathy (DCM), × 200.

## Data Availability

The datasets generated during and/or analysed during the current study are available from the corresponding author on reasonable request.

## Abbreviations

ARVC: Arrhythmogenic right ventricular cardiomyopathy
CI: Confidence interval
CMR: Cardiac magnetic resonance imaging
DCM: Dilated cardiomyopathy
ECG: Electrocardiogram
EF: Ejection fraction
EMB: Endomyocardial biopsy
HCM: Hypertrophic cardiomyopathy
ILW: Infero-lateral wall
IRB: Institutional review board
IVS: Interventricular septum
LA: Left atrium
LAI: Left atrium index
LGE: Late gadolinium enhancement
LV: Left ventricle
LVEDD: Left ventricular end-diastolic diameter
LVEDV: Left ventricular end-diastolic volume
LVEDVI: Left ventricular end-diastolic volume index
LVEF: Left ventricular ejection fraction
LVESV: Left ventricular end-systolic volume
LVGFI: Left ventricular global function index
LVMM: Left ventricular myocardial mass
LVMMI: Left ventricular myocardial mass index
LVSV: Left ventricular stroke volume
MRI: Magnetic resonance imaging
RA: Right atrium
RAI: Right atrium index
RV: Right ventricle
RVEDV: Right ventricular end-diastolic volume
RVEDVI: Right ventricular end-diastolic volume index
RVEF: Right ventricular ejection fraction
RVESV: Right ventricular end-systolic volume
RVSV: Right ventricular stroke volume
SA: Short axis
SCD: Sudden cardiac death
SD: Standard deviation
SSFP: Steady state free precession
WMA: Wall motion abnormalities
