# Supplementary figures and images for: Cardiac MRI findings to differentiate athlete's heart from hypertrophic (HCM), arrhythmogenic right ventricular (ARVC) and dilated (DCM) cardiomyopathy
Source: Int J Cardiovasc Imaging. 2021 May 21;37(8):2501–15. doi: 10.1007/s10554-021-02280-6 (PMC8302518; doi:10.1007/s10554-021-02280-6)

## Slide 1
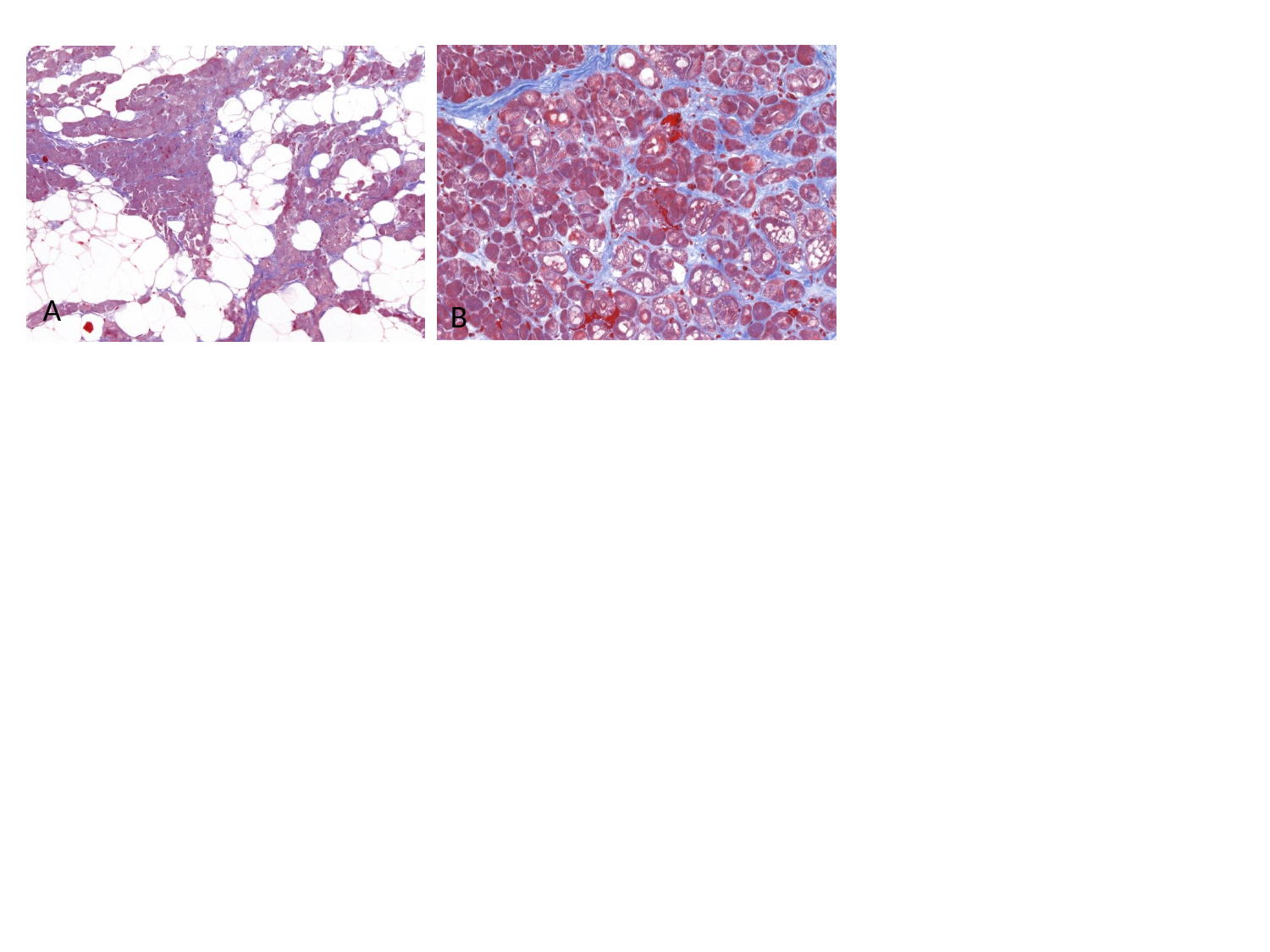

A
B

Supplement: Supplementary file 1 — Supplementary file1 (PPTX 442 kb) Figure 5 (Supplemental): Visualization of myocardial damage in endomyocardial biopsies by Masson Trichrome staining. (A) Arrhythmogenic right ventricular cardiomyopathy, × 100, (B) Dilated cardiomyopathy (DCM), × 200. [file 10554_2021_2280_MOESM1_ESM.pptx]
